# Novel Platinum(II) Tetrazine Complex Capable of Live‐Cell IEDDA Reaction

**DOI:** 10.1002/cbic.202500376

**Published:** 2025-08-29

**Authors:** Paul D. O’Dowd, Dan Wu, Alby Benny, Ellen King, Alice Harford, Brendan Twamley, Olga Piskareva, Donal F. O’Shea, Darren M. Griffith

**Affiliations:** ^1^ Department of Chemistry Royal College of Surgeons in Ireland Dublin 2 D02 YN77 Ireland; ^2^ SSPC The Science Foundation Ireland Research Centre for Pharmaceuticals Ireland; ^3^ Cancer Bioengineering Group & Tissue Engineering Research Group (TERG) Department of Anatomy and Regenerative Medicine RCSI University of Medicine and Health Sciences Dublin D02 YN77 Ireland; ^4^ School of Pharmacy and Biomolecular Sciences RCSI University of Medicine and Health Sciences Dublin D02 YN77 Ireland; ^5^ School of Chemical and BioPharmaceutical Sciences TU Dublin Grangegorman Dublin D07 H6K8 Ireland; ^6^ Department of Chemistry Trinity College Dublin Dublin 2 D02 PN40 Ireland; ^7^ Advanced Materials and Bioengineering Research Centre (AMBER) RCSI University of Medicine and Health Sciences and Trinity College Dublin Dublin D02 YN77 Ireland

**Keywords:** FLIM, fluorescence, IEDDA, platinum, tetrazine

## Abstract

The development of the first Pt(II) tetrazine complex, *trans‐*[Pt(II)Cl_2_(dmso)(CH_3_‐Tz‐Bz‐NH_2_)] (**1**), is reported, which exhibits good in vitro cytotoxicity against MDA‐MB‐231 cells and succesfully undergoes inverse electron demand Diels–Alder (IEDDA) reactions with *trans*‐cyclooctene (TCO) and bicyclononyne (BCN) derivates in solution. We demonstrate a live‐cell IEDDA reaction of **1** with a BF_2_‐azadipyrromethene fluorophore (NIR‐AZA) posessing a BCN handle. A live‐cell bioorthogonal reaction is established using fluorescence lifetime imaging microscopy (FLIM), through a fluorescence lifetime change of 0.3 ns from BF_2_‐azadipyrromethene fluorophore starting material to IEDDA Pt fluorophore reaction product. As there is a distinct difference in fluorescence lifetimes between starting material and product, this approach removes the necessity for designing challenging off to on fluorogenic Pt probes and washing steps when developing bioorthogonal cell‐imaging strategies for Pt complexes.

## Graphical abstract

The first Pt(II) tetrazine complex, *trans*‐[PtCl_2_(dmso)(CH_3_‐Tz‐Bz‐NH_2_)] (1), is reported, showing good cytotoxicity against MDA‐MB‐231 cells and undergoing IEDDA reactions with TCO and BCN derivatives. A live‐cell IEDDA reaction in MDA‐MB‐231 cells with a BCN‐bearing BF_2_‐azadipyrromethene fluorophore is tracked via FLIM, owing to a 0.3 ns lifetime shift.

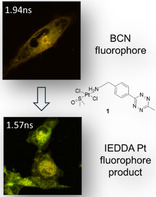

## 1 Introduction

**Figure 1:**
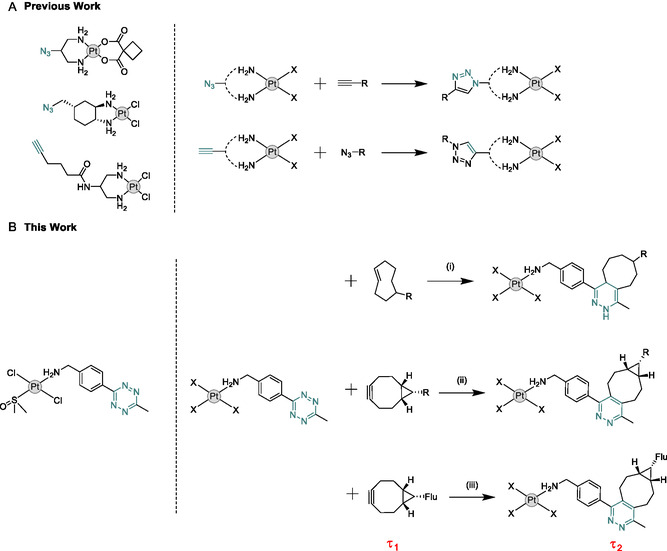
A) Representative examples of Pt(II) click templates developed to date, which possess alkyne or azide ligand‐based click handles^[^ ^5^ ^]^ and B) IEDDA Pt(II) tetrazine template described in this study; 1) IEDDA with TCO derivative; 2) IEDDA with BCN derivative; and 3) IEDDA with BCN fluorescent dye monitored by FLIM.

Platinum (Pt)‐based anticancer drugs, such as cisplatin, carboplatin, and oxaliplatin, remain an important class of chemotherapeutic used clinically to treat many solid tumors. The cytotoxicity of Pt anticancer drugs is primarily associated with the formation of DNA adducts, which activate DNA damage responses and ultimately programmed cell death through apoptosis. Despite this, it is well known that Pt(II) centers readily react with a range of nucleophiles inside the body, such as mitochondrial DNA, RNA, as well as multiple mitochondrial and extramitochondrial proteins.^[^ ^1^ ^]^ As such, there is great interest in elucidating alternative or additional mechanisms of action for Pt(II) centers. Additionally, despite the clinical success of anticancer Pt(II) drugs, their efficacy is limited by toxic side effects^[^ ^2^ ^]^ and resistance.^[^ ^3^ ^]^ Our group has an interest in exploiting click chemistry‐based strategies to address some of these issues.^[^ ^4^ ^]^ Click chemistry readily facilitates the conjugation of Pt complexes with, for example, secondary chemotherapeutics, targeting moieties/delivery systems, and fluorescent reporters (pre and post‐treatment labeling).^[^ ^4^ ^e]^ A variety of Pt(II) click templates have been developed over the years, which possess alkyne or azide click handles for reaction through CuAAC or SPAAC click reactions, **Figure** **1**.^[^ ^5^ ^]^

More recently, attention in chemical biology has pivoted toward bioorthogonal reactions and in particular inverse electron demand Diels–Alder (IEDDA) reactions. In an IEDDA reaction an electron‐poor, such as a 1,2,4,5‐tetrazine, reacts with an electron‐rich dienophile, such as an alkene or alkyne, to form a new six‐membered ring in a *π*4s + *π*2s fashion.^[^ ^6^ ^]^ These reactions are biocompatible, catalyst‐free, fast and selective, and have been successfully employed for bioorthogonal labeling and cellular tracking applications.^[^ ^7^ ^]^ To date there are no examples of Pt complexes possessing tetrazine click‐handles or that can undergo IEDDA reactions, in the literature.

There is considerable interest in the development of Pt‐based probes and tracking the cellular uptake and localization of Pt anticancer complexes using fluorescence microscopy.^[^ ^4^ ^a,c−e,^ ^5^ ^a,b,d,^ ^8^ ^]^ Fluorescence microscopy offers many advantages over X‐ray fluorescence (XRF) imaging for example, which can be used to detect elements in cells, such as being more suitable for live‐cell and dynamic biological studies, having higher sensitivity, and is highly customizable given the array of available fluorophores.^[^ ^9^ ^]^

In general fluorogenic‐responsive bioorthogonal reactions, e.g. azide–alkyne dipolar or IEDDA, rely on fluorogenic triggers such as conversion of azides to triazoles, and tetrazines to pyridazines, which produce excited state changes to the fluorophore. In live cell imaging there may be a requirement for washing steps to remove unreacted fluorophore or a challenge achieving a suitable off to on fluorescence response on successful bioorthogonal reaction.^[^ ^10^ ^]^ Significantly fluorescence lifetime imaging microscopy (FLIM) can be employed to monitor bioorthogonal reactions by tracking changes in the fluorescence lifetime of a fluorescent probe as it reacts in real‐time in cells.^[^ ^11^ ^]^

We report the development of a Pt complex containing a 1,2,4,5‐tetrazine ligand which acts as a bioorthogonal click handle. Herein we report the synthesis, characterization, and in vitro cytotoxicity of *trans*‐[Pt(II)Cl_2_(dmso)(CH_3_‐Tz‐Bz‐NH_2_)] **1,** where CH_3_‐Tz‐Bz‐NH_2_ is 4‐(6‐Methyl‐1,2,4,5‐tetrazin‐3‐yl)phenyl)methan‐amine or methyl tetrazine amine. Furthermore we describe successful in solution IEDDA reactions with BCN and TCO derivatives as well as demonstrating a live‐cell IEDDA reaction with a BF_2_‐azadipyrromethene fluorophore (NIR‐AZA) posessing a BCN handle using FLIM.

## 2 Results and Discussion

### 2.1 Synthesis and Characterization

In this work we set out to develop the first example of a Pt complex containing a 1,2,4,5‐tetrazine ligand, which can be further functionalized through IEDDA click chemistry. For this, we selected methyl tetrazine amine (CH_3_–Tz–Bz–NH_2_) as our N‐donor tetrazine ligand as it offered the desired balance between reactivity and stability in aqueous solution.^[^ ^7^ ^]^

**Scheme 1:**
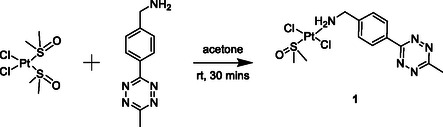
Synthesis of *trans*‐[Pt(II)Cl_2_(dmso)(CH_3_‐tz‐bz‐NH_2_)] **1** from *cis‐*[Pt(II)Cl_2_(dmso)_2_].

Tetrazine‐based N‐donor ligands would likely be unstable over the course of the reaction times associated with the synthesis of classical Pt(II) complexes in aqueous solutions.^[^ ^7^ ^]^ We therefore sought to synthesize a *trans*‐[PtCl_2_(dmso)(N‐donor)] type complex as the reactions can be undertaken in nonaqueous solvents such as acetone ensuring our desired product can be obtained in high purity and yield. Reaction of CH_3_–Tz–Bz–NH_2_ with the platinum precursor *cis*‐[Pt(II)Cl_2_(dmso)_2_] in acetone afforded the desired complex, *trans‐*[Pt(II)Cl_2_(dmso)(CH_3_‐Tz‐Bz‐NH_2_)] (**1**) in good yield, **Scheme** **1**.

**1** was fully characterized by ^1^H and ^13^C nuclear magnetic resonance (NMR) spectroscopy, and high‐resolution mass spectrometry (HRMS), with purity confirmed through elemental analysis, (Figure S1–S3, Supporting Information). Briefly the elemental analysis was fully consistent with two chlorido ligands, one dmso ligand and one methyl tetrazine amine ligand per Pt(II) center. In the ^1^H NMR spectrum of **1** (CDCl_3_, Figure S1, Supporting Information), the four aromatic protons associated with the benzene ring of methyl tetrazine amine for example are observed as two doublets at 7.58–8.59 ppm. The amino and methylene protons are both found as triplets at 4.71 and 4.16 ppm respectively and the methyl protons as a singlet at 3.10 ppm. The singlet at 3.44 ppm is attributed to the six protons of the coordinated dmso ligand. In the ^13^C NMR spectrum of **1** (Figure S2, Supporting Information), the aromatic carbons of the benzene ring are found between 128–132 ppm and the signal for the coordinated dmso methyl carbons at 44 ppm. In the HRMS (ESI^+^, MeOH) spectrum [M + Na]^+^ is observed at 567.0088 amu and [M + K]^+^ at 592.9828 amu with the expected isotopic pattern, (Figure S3, Supporting Information).

### 2.2 X‐ray Crystallography

**Figure 2:**
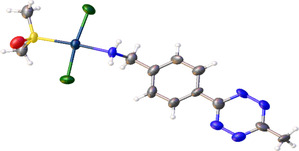
View of one of the two independent disordered molecules in the asymmetric unit of 1 at 84% occupancy, showing the *trans* conformation of the structure. Atomic displacement shown at 50% probability. See Figure S4 and S5, Supporting Information for the full asymmetric unit.

Single crystals of **1** were obtained on standing a saturated solution of **1** in acetone at 4 °C. X‐ray crystallography of **1** confirmed successful coordination of the methyl amine tetrazine ligand to platinum as well as the *trans*‐configuration of **1**, **Figure** **2**.

### 2.3 Isomerization and Stability of 1 in Solution

The *trans*‐[Pt(II)Cl_2_(dmso)L] type complexes, where *L* is a planar N‐donor aromatic ligand or heterocyclic ligand, which are similar in overall structure to **1,** have previously been shown to isomerize in solution.^[^ ^12^ ^]^ For instance, Farrell and coworkers demonstrated the isomerization of *trans‐*[Pt(II)Cl_2_(dmso)(quinoline)] to *cis‐*[Pt(II)Cl_2_(dmso) (quinoline)] in DMSO.^[^ ^12^ ^a]^ To explore whether **1** shows a similar isomerization profile in solution**, 1** was dissolved in *d*‐DMSO and ^1^H NMR spectra recorded over the course of 3 days. Results from this experiment showed conversion of the *trans*‐ to *cis* *‐*isomer in solution over the course of 24 h, with an approximate 74:26 (*cis*:*trans*) ratio noted, while further conversion beyond 24 h was limited, (Figure S6, Supporting Information). Therefore, it is clear that any bioactivity associated with *trans*‐[Pt(II)Cl_2_(dmso)L] type complexes should be attributed to a mix of the *trans*‐ and *cis*‐isomers with the concentration of the *cis*‐isomer increasing over time.

A UV–vis study was undertaken to gain an insight into the stability of **1** in cell media. **1** is principally stable in DMEM:DMSO (200:1) (Figure S7, Supporting Information) up to 8 h, though a notable bathochromic shift is observed from 8 to 24 h.

### 2.4 In Vitro Cytotoxicity

**Figure 3:**
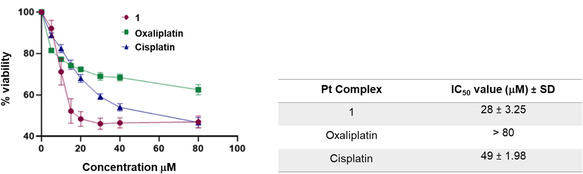
Dose‐response plots and table of IC_50_ values for **1**, oxaliplatin and cisplatin against MDA‐MB‐231 cell line at 72 h treatment.

The *trans*‐[Pt(II)Cl_2_(dmso)L] type complexes have been reported to exhibit appreciable anticancer activity, though in most cases inferior activity as compared to cisplatin.^[^ ^12^ ^b,^ ^13^ ^]^ The in vitro cytotoxicity of **1** was determined in the triple‐negative breast cancer cell line MDA‐MB‐231 at a 72 h timepoint. Significantly **1** exhibited enhanced activity, with an IC_50_ of 28 µM, as compared to the IC_50_ values obtained for cisplatin (49 µM) and oxaliplatin (>80 µM), **Figure** **3**.

### 2.5 1 Successfully Undergoes IEDDA Click Modification

**Scheme 2:**
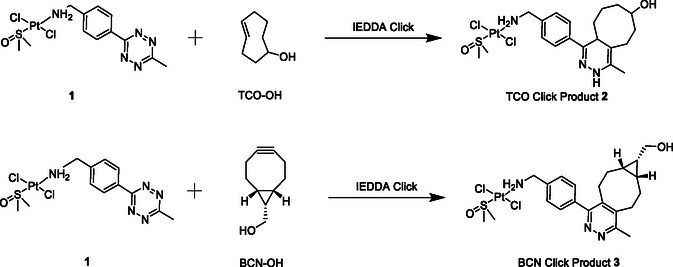
IEDDA reactions of **1** with TCO and BCN derivatives.

Tetrazines readily react with TCO and BCN. To ensure Pt tetrazine complex **1** could successfully be modified through IEDDA reactions, the complex was reacted at RT separately with both (*E*)‐Cyclooct‐4‐enol (TCO‐OH) in CDCl_3_ for 30 min and (1*R*, 8*S*,9*S*)‐Bicyclo[6.1.0]non‐4‐yn‐9‐ylmethanol (BCN‐OH) in *N*,*N*‐dimethyl‐formamide‐d7 for 2 h. Reaction progress was monitored by ^1^H NMR spectroscopy and HRMS **Scheme** **2**.

The disappearance of signals corresponding to the starting material **1** together with the emergence of new diagnostic signals as evidenced in the stacked ^1^H NMR spectra, (Figure S8 and S10, Supporting Information). supports formation of **2** and **3.** For instance in both reactions there is a clear upfield shift of the aromatic protons and methyl protons in the CH_3_–Tz–Bz–NH_2_ ligand on IEDDA reaction. Formation of the desired products in solution was confirmed by the HRMS data; [M + H]^+^ (643.1086 amu), [M + Na]^+^ (665.0993 amu), and [M + K]^+^ (681.0754 amu) for the TCO‐OH reaction and [M + H]^+^ (667.1551 amu) and [M + Na]^+^ (689.1158 amu) for the BCN‐OH reaction, (Figure S9 and S11, Supporting Information). These results demonstrate the desired IEDDA product is formed in both cases. As such, these results indicate, **1** can successfully undergo in solution IEDDA click reactions with TCO and BCN derivatives, demonstrating it can be functionalized with TCO or BCN appended fluorophores via an IEDDA reaction.

Pseudo‐first order reaction rates were measured and relative rates calculated for the IEDDA reactions between 1) methyl tetrazine amine and BCN–OH and TCO–OH and 2) complex **1** and BCN–OH and TCO–OH at room temperature (Figure S12 and Table S3, Supporting Information). As expected the IEDDA reaction rates were faster for reactions where the dienophile was TCO–OH. Reaction of methyl tetrazine amine for example with TCO–OH was 23 times faster than the reaction of methyl tetrazine amine with BCN–OH. Coordination of the Pt(II) center to methyl tetrazine amine in complex **1** had little influence on the IEDDA reaction kinetics. For instance the relative rates of methyl tetrazine amine with BCN–OH, which was set as 1, correlates more or less with the calculated relative rate of IEDDA reaction of 0.9 for complex **1** with BCN–OH. Similarly relative IEDDA reaction rates for methylamine tetrazine and complex **1** with TCO–OH were 23 and 21, respectively.

### 2.6 Live Cell IEDDA Reaction with BF_2_‐Azadipyrromethene Fluorophore Possessing a BCN Handle

**Scheme 3:**
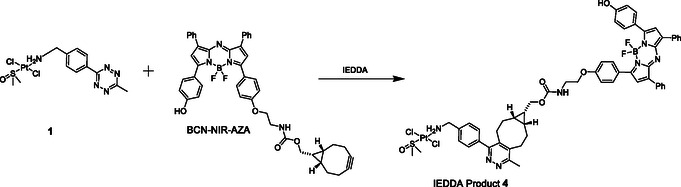
IEDDA reaction of **1** with BCN–NIR–AZA to give IEDDA product **4**.

We selected a BF_2_‐azadipyrromethene fluorophore with a BCN handle (BCN‐NIR‐AZA)^[^ ^14^ ^]^ for our live cell IEDDA reaction with complex **1**. NIR‐AZA fluorophores as a class are generally robust and readily synthesized.^[^ ^15^ ^]^ Significantly their fluorescence wavelengths in the near‐infrared range of 650–850 nm, are ideally suited for imaging within living systems.^[^ ^16^ ^]^ Reaction of 1 with BCN‐NIR‐AZA in CDCl_3_ gave IEDDA product **4**, **Scheme** **3**. The reaction was monitored via ^1^H NMR spectroscopy with the reaction essentially complete after 30 min, (Figure S13, Supporting Information). Formation of the desired product was also evidenced by the HRMS data, [M + H]^+^ (1265.3195 amu) and [M + Na]^+^ (1287.3042 amu), Figure S14, Supporting Information.

**Figure 4:**
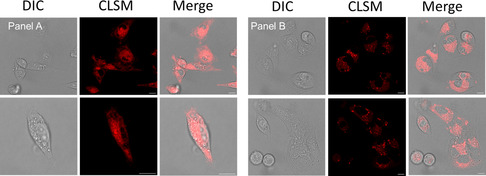
Live cell IEDDA reaction of **1** with BCN‐NIR‐AZA to give IEDDA product. Left panel A: fluorescence imaging after 1 h. Right panel B: fluorescence imaging after 4 h. Scale bar: 10 µm.

**Figure 5:**
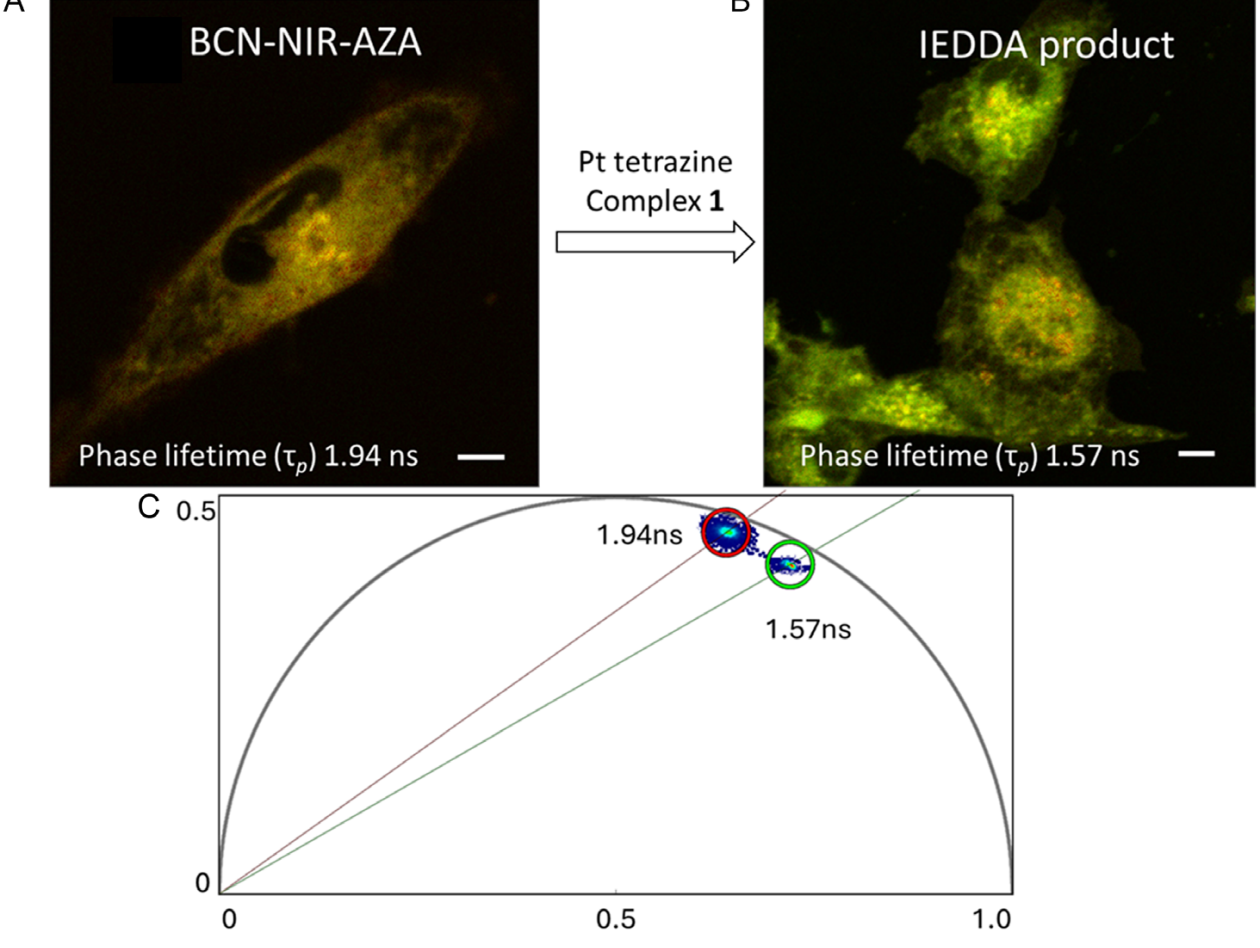
Phasor analyzed FLIM imaging of biorthogonal reaction of BCN‐NIR‐AZA (10 μM) with Pt tetrazine complex **1** (25 μM) in live MDA‐MB‐231 cells. A) Phasor mapped FLIM image showing cells following 1 h incubation with BCN‐NIR‐AZA. B) Phasor mapped FLIM image showing cells pretreated with BCN‐NIR‐AZA for 1 h followed by treatment with **1** for 1 h resulting in transformation into IEDDA product. C) Phasor plot of intracellular IEDDA product (green circle, phase lifetime 1.57 ns) formed via biorthogonal reaction in comparison to BCN‐NIR‐AZA (red circle, phase lifetime 1.94 ns). Scale bar: 5 µm.

The potential for **1** to undergo IEDDA transformations in vitro was next investigated in live cells. MDA‐MB‐231 cells were pretreated with 10 μM BCN–NIR–AZA for 1 h, followed by treatment with **1** (25 μM). Cells were subsequently imaged at 1 and 4 h using confocal laser scanning microscopy (CLSM) and FLIM. The CLSM images showed effective internalization (**Figure** **4**). The intracellular localization, which was primarily in the cytosol at 1 h, remains evident after 4 h. FLIM offers a distinct advantage for bioorthogonal imaging as the excited‐state lifetimes of multiple fluorophores within different cellular compartments can be acquired simultaneously. The phasor analysis of FLIM images allows a straightforward interpretation of these fluorescence signals and we sought to apply this to observe the intracellular reaction of BCN–NIR–AZA and complex **1**. Phasor transformation of live cell FLIM images following incubation with BCN–NIR–AZA only (**Figure** **5**) showed the phase lifetime of 1.9 ns. Significantly it was observed that cells first treated with BCN–NIR–AZA, to which complex **1** was subsequently added for 1 h, showed a change in intracellular phase lifetime to 1.6 ns (Figure 5). The successful live‐cell bioorthogonal reaction was therefore established using FLIM, through a fluorescence lifetime change of 0.3 ns from BF_2_‐azadipyrromethene fluorophore starting material to IEDDA Pt fluorophore reaction product.

The FLIM lifetimes in MDA‐MB‐231 cells of 1) the in‐cell IEDDA product between the methyl tetrazine amine and the BCN–NIR–AZA and 2) the “preclicked” product **4** were measured as controls. The lifetime of the IEDDA product between the methyl tetrazine amine and the BCN–NIR–AZA (Figure S17, Supporting Information) was found to be 1.8 ns, which differs significantly from the FLIM lifetime of 1.6 ns measured for the in‐cell IEDDA product of complex **1** with BCN–NIR–AZA and also the FLIM lifetime of the “preclicked” IEDDA product **4** of 1.6 ns (Figure S18, Supporting Information).

## 3 Conclusion

To conclude, herein we report the development of the first Pt(II) tetrazine complex, **1**, which exhibits good in vitro cytotoxicity against MDA‐MB‐231 cells and succesfully undergoes in solution IEDDA reactions with TCO and BCN derivatives. We demonstrate a live‐cell IEDDA reaction of **1** with a BF_2_‐azadipyrromethene fluorophore posessing a BCN handle. The progress of the live‐cell bioorthogonal reaction was successfully tracked, using FLIM, through a fluorescence lifetime change of 0.3 ns from BF_2_‐azadipyrromethene fluorophore starting material to IEDDA Pt fluorophore reaction product **4**. As there is a distinct difference in fluorescence lifetimes between starting material and product, this approach removes the necessity for designing challenging off to on fluorogenic Pt probes and washing steps when developing bioorthogonal cell‐imaging strategies for Pt complexes. Complex **1** expands the range of Pt(II) templates available for the design of multifunctional Pt complexes and highlights that tetrazine based platforms and FLIM have a role to play in future studies into the mechanisms of action and tracking of Pt‐based drugs.

## Supporting Information

The authors have cited additional references within the Supporting Information.

## Conflict of Interest

The authors declare no conflict of interest.

## Supporting information

Supplementary Material

## Data Availability

The data that support the findings of this study are available from the corresponding author upon reasonable request.
