# Supplementary material for: Novel Platinum(II) Tetrazine Complex Capable of Live‐Cell IEDDA Reaction
Source: Chembiochem. 2025 Aug 29;26(18):e202500376. doi: 10.1002/cbic.202500376 (PMC12447375; doi:10.1002/cbic.202500376)
Supplement: Supplementary file 1 — Supplementary Material [file CBIC-26-e202500376-s001.pdf]

Novel Platinum(II) tetrazine complex capable of live-cell IEDDA reaction

Paul O'Dowd, Dan Wu, Alby Benny, Ellen King, Alice Harford, Brendan Twamley, Olga Piskareva, Donal F. O'Shea, and Darren M. Griffith

Supplementary Information

Materials & Instrumentation

All chemicals and solvents were purchased from Sigma Aldrich (*Sigma Aldrich Ireland Ltd, Co. Wicklow, Ireland*) unless stated otherwise, and were used without further purification. ^{1}H NMR and ^{13}C NMR spectra were recorded on a Bruker Avance 400 MHz NMR spectrometer. All spectra were analysed using MestReNova software and the residual undeuterated solvent signals were used as internal references. High resolution electrospray ionization (ESI) mass spectrometry was carried out in positive mode on a Bruker Compact™ mass spectrometer and analysed using mMass software. High performance liquid chromatography was performed with a Shimazdu SIL-20AHT HPLC instrument equipped with a Shimazdu SPD-20AV prominence UV/Vis detector.

Syntheses

*c*is-[Pt(DMSO)$_2$(Cl)$_2$] and 4-(6-Methyl-1,2,4,5-tetrazin-3-yl)phenyl)methan-amine (CH_3-Tz-Bz-NH_2) were synthesised as previously reported. [1, 2]

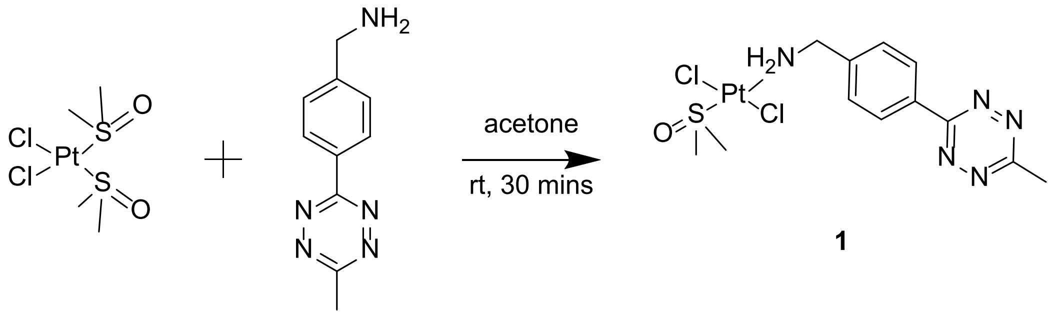

1

Synthesis of *trans*-[Pt(II)Cl$_2$(dmso) (CH_3-Tz-Bz-NH_2)] (1)

cis-[PtCl$_2$(dmso)$_2$] (250 mg, 0.59 mmol) was suspended in acetone (20 mL). To this was added CH_3-Tz-Bz-NH_2 as the freebase (119 mg, 0.59 mmol) in acetone (5 mL). The resulting mixture was stirred for 30 minutes at room temperature and then the volume reduced to approx. 5 mL resulting in the formation of a red precipitate. This precipitate was isolated by vacuum filtration to yield a crystalline red solid. (205.9 mg, 63.8%) Crystals suitable for single crystal X-ray diffraction analysis were isolated by leaving the filtrate stand at 4 °C overnight. ^{1}H NMR (400 MHz, $CDCl_3$) δ 8.57 (d, 2H, 8.3 Hz), 7.56 (d, 2H, 8.3 Hz), 4.85-4.61 (m, 2H), 4.24-

4.10 (m, 2H), 3.37 (s, 6H), 3.06 (s, 3H). ^{13}C NMR (101 MHz, $CDCl_3$) δ: 167.5, 163.9, 141.6, 132.0, 129.2, 128.8, 49.6, 44.0, 21.3. HRMS (ESI$^+$) (MeOH) [M + Na]$^+$: $C_{12}H_{17}Cl_2N_5NaOPt$, calc.: 567.0076, found: 567.0088; [M + K]$^+$: $C_{12}H_{17}Cl_2KN_5OPt$, calc.: 592.9816, found: 592.9828. Elem. Anal. Calcd for $C_{12}H_{17}Cl_2N_5OPt$: C, 26.43; H, 3.14; N, 12.84; Cl, 13.00. Found: C, 26.59; H, 2.92; N, 12.50; Cl, 13.20.

Cell Culture and in vitro assay

Cell suspensions containing 1x10^4 cells/ml were seeded into to 96 well plates and incubated overnight at 37°C and 5% CO_2. The next day, cytotoxic drug dilutions at their final concentration in cell culture medium were added to each well and incubated for 72 hrs at 37°C and 5% CO_2.

Assessment of cell survival in the presence of test Pt complexes was determined by the acid phosphatase assay. Cells were washed with pre-warmed PBS and 100 µl of freshly prepared phosphatase substrate (10 mM p-nitrophenol phosphate (Sigma)) in 0.1M sodium acetate (Sigma), 0.1% triton X-100 (BDH), pH 5.5 was added to each well. The plates were wrapped in tinfoil and incubated in the dark at 37°C, 5% CO_2 for 2 hr. The enzymatic reaction was stopped by the addition of 50 µl of 1M NaOH (Sigma). The plate was read in a VICTOR™ X dual beam plate reader (Perkin Elmer) at 405 nm with a reference wavelength of 620 nm. Cell proliferation was represented as growth relative to the negative control cells. The number of biological replicates = 3. Statistical significance was determined by a 2-tailed t-test. The IC_{50} values of the drug was determined from a plot of the percentage survival (relative to the control cells) versus cytotoxic test Pt complex concentration.

IEDDA kinetics

IEDDA reaction kinetics were measured using a ThermoFischer Genesys 150 Spectrophotometer. 1 mM stock solutions of CH_3-Tz-Bz-NH_2 (methyl tetrazine amine ligand) and *trans*-[Pt(II)Cl_2(dmso)(CH_3-Tz-Bz-NH_2)] (1) were prepared in DMF. 5 mM stock solutions of ((1R,8S,9s)-bicyclo[6.1.0]non-4-yn-9-yl)methanol (BCN-OH) and (E)-cyclooct-4-en-1-ol (TCO-OH) in DMF were prepared. The rate of IEDDA reaction was measured by mixing stock solutions of each tetrazine compound (250 µL) and either BCN-OH (250 µL) or TCO-OH (250 µL) in a quartz cuvette at room temperature and monitoring the reduction of the tetrazine absorbance (λ = 542 nm for methyl tetrazine amine ligand and λ = 538 nm for complex 1). The absorbance (A) was measured at 5 min intervals for 60 min for the reaction with BCN-OH and at 6 second intervals for 2 min for the reaction with TCO-OH. The reactions were carried out in triplicate. The pseudo first-order observed rate consants (kobs, s^{-1}) were then calculated from the slope of the linear regression fitting of ln(A) versus time.

Microscopy

CLSM and FLIM images were acquired on a Leica Stellaris 8 Falcon microscope fitted with NKT Photonics White Light Laser (440 nm-790 nm) and controlled by LAS X (version. 4.4.0.24861). Live cell imaging experiments were carried out under Okolab incubation system to maintain the temperature at 37 °C and CO_2 at 5%. Images were acquired using a Leica HC PL APO CS2 100X/1.40 oil immersion objective. Excitation wavelength was obtained using the White Light Laser (WLL) tuned at 491 nm. A HyD detector was used to collect emission in the range 680nm-750nm for BCN-NIR-AZA fluorophore emission. DAPI channel and Bright field channels were also activated.

The FLIM function was used to acquire the real time fluorescence lifetime imaging in the cells. A mono-exponential/1 component fit was used to give the best fitting curve and to extract the lifetimes at different time points. Phasor plots analysis were processed by LAS X Falcon software, for a better visualization of the changes in the lifetime. A HyD detector was used for the collection.

Crystallography

A clear red, needle-shaped crystal was mounted on a MiTeGen micromount with glue. Data for **1** were collected from a single crystal at 296(2) K on a Bruker APEX2 Kappa Duo Kappa diffractometer with a microfocus sealed X-ray tube using mirror optics as a monochromator and an APEX2 detector. The diffractometer was equipped with a Cobra (Oxford Cryosystems Ltd, Oxford, UK) low temperature device and used Cu K_α radiation (λ = 1.54178 Å). All data were integrated with SAINT V8.40B and a multi-scan absorption correction using SADABS 2016/2 was applied.[3,4] The structure was solved by dual methods with SHELXT v2018/2 and refined by full-matrix least-squares methods against F^2 using SHELXL v2019/3.[5,6] All non-hydrogen atoms were refined with anisotropic displacement parameters. All hydrogen atoms were refined isotropic on calculated positions using a riding model with their U_{iso} values constrained to 1.5 times the U_{eq} of their pivot atoms for terminal sp^3 carbon atoms and 1.2 times for all other carbon atoms. Disordered moieties were refined using bond lengths restraints and displacement parameter restraints. Crystallographic data for the structure (CCDC no. 2449082) reported in this paper have been deposited with the Cambridge Crystallographic Data Centre.[7] The data can be obtained free of charge from The Cambridge Crystallographic Data Centre via www.ccdc.cam.ac.uk/structures. This report was generated using FinalCif.[8]

Refinement details for Pt-tetrazine

Data collected at room temperature using a Cu microfocus source. Very little diffraction was seen using low temperature. The structure at RT was modelled disordered - at least two positions for the pair of molecules per asymmetric unit. The large residuals are possibly absorption artefacts (Cu radiation) or another Pt centre/disordered site. At 3 electrons, this is approx. 4% of a Pt. Other potential atoms sites are not observable given the data quality at RT. Disorder in Pt1/P1b, 84:16% occupied; Pt2/Pt2b also 84:16% occupied and modelled with geometric (DFIX, SADI, FLAT) and displacement (SIMU) restraints and constraints (EADP). Ring system were modelled as rigid hexagons (AFIX) and minor components were modelled as entire rigid groups. Refined as an inversion twin with a Flack parameter of 0.47(6).

Characterisation of Pt-tetrazine 1:

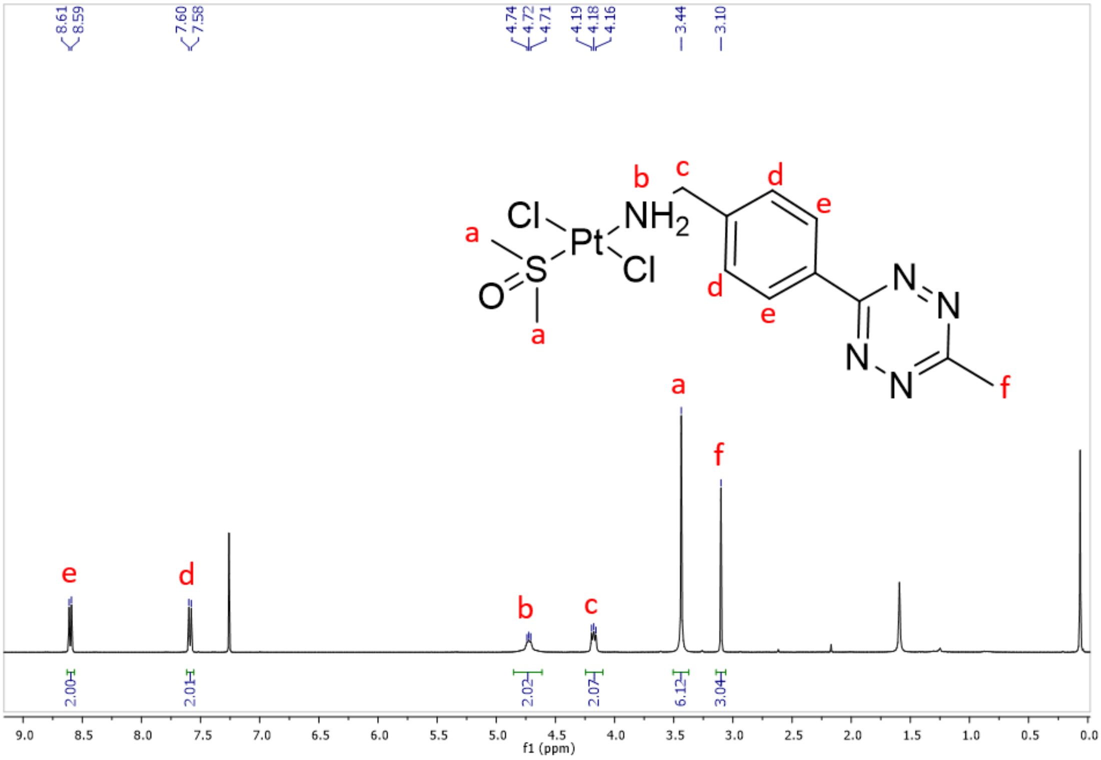

Figure S1: ^{1}H NMR of Pt-tetrazine in $CDCl_3$

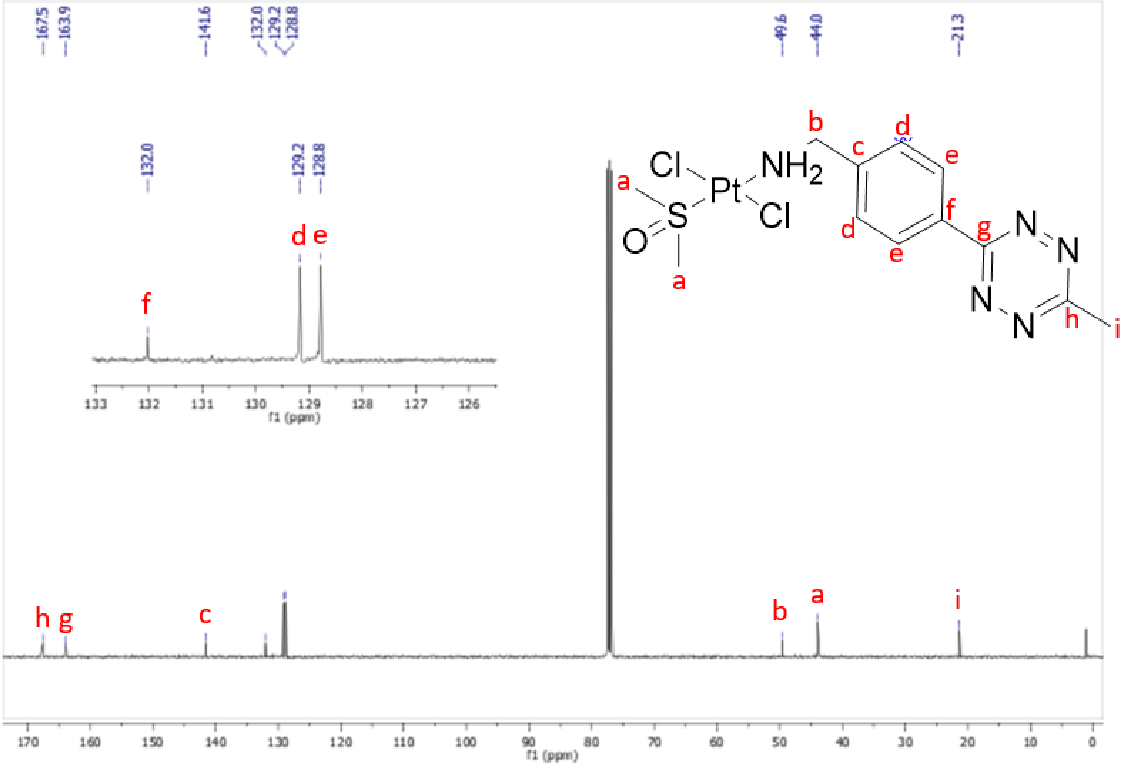

Figure S2: ^{13}C NMR of Pt-tetrazine in $CDCl_3$

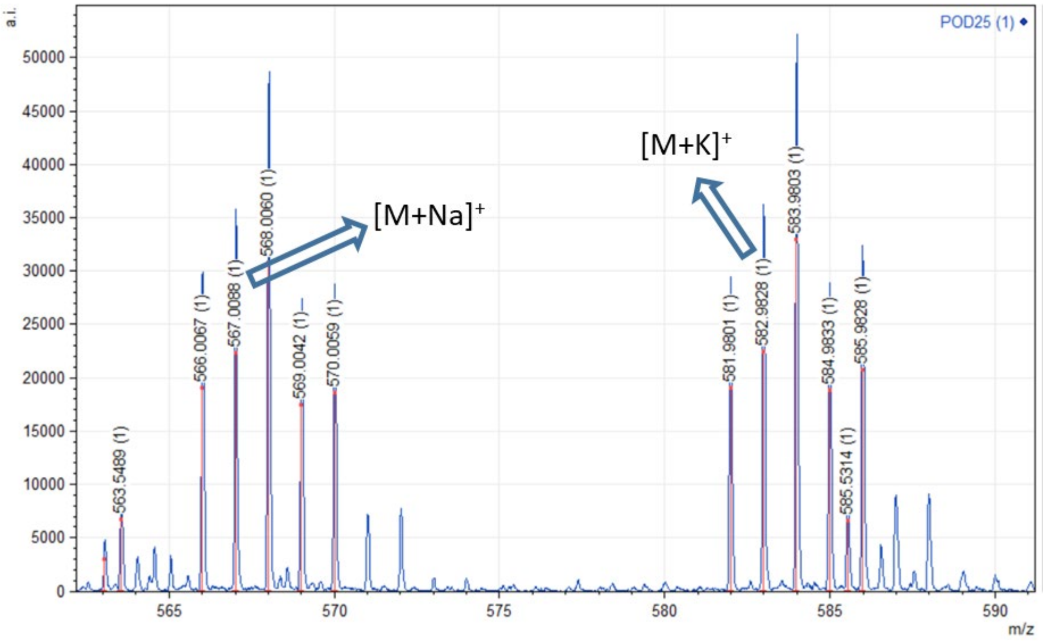

Figure S3: HRMS (ESI)$^+$ Spectrum of Pt-tetrazine in MeOH

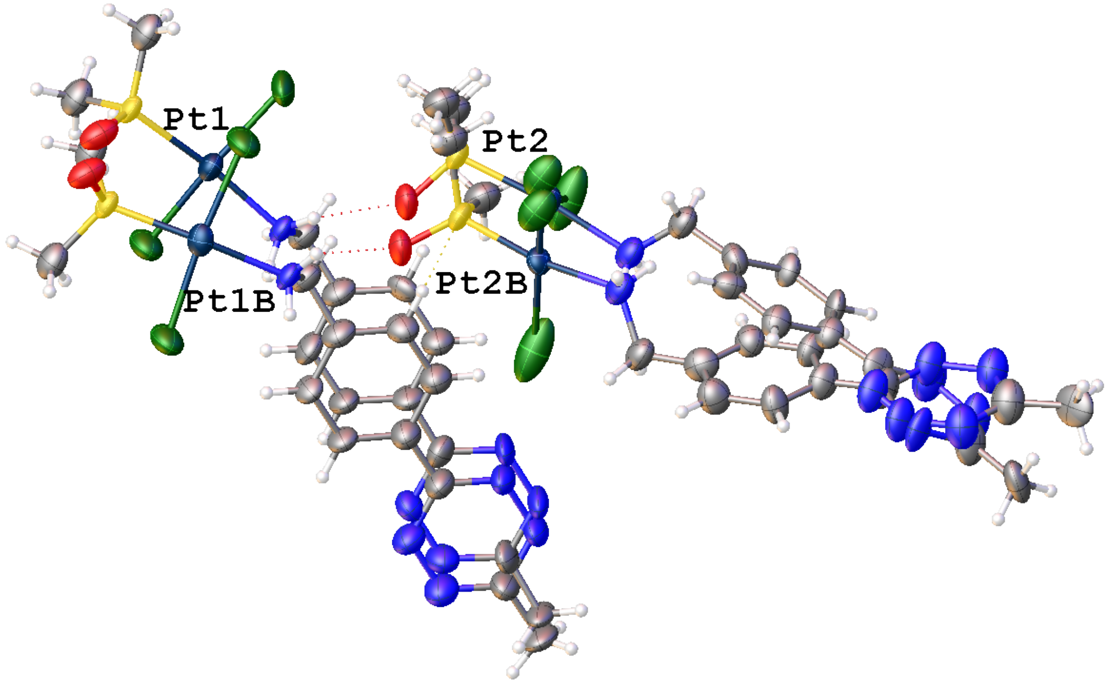

Figure S4. A view of the complete asymmetric unit in **1**, showing the two disordered independent molecules. The individual moieties are shown below in Figure S5. Major occupancy, 84%, minor occupancy 16%. Displacement shown at 50% probability and only the metal centre is labelled for clarity.

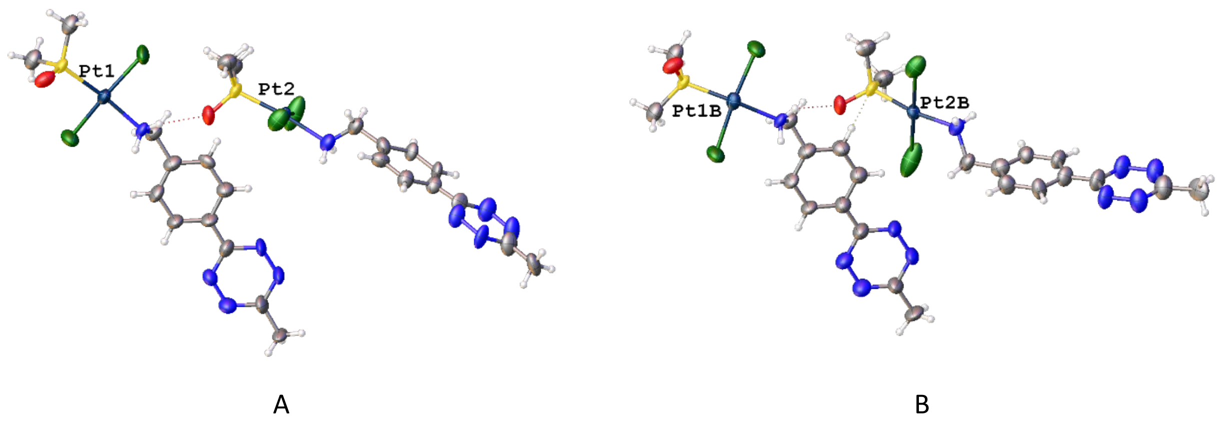

Figure S5. Separate representations of each disordered moiety in the asymmetric unit with (A) major occupied moiety 84% and (B) minor occupied moiety 16%. Displacement shown at 50% probability and only the metal centre is labelled for clarity.

Table S1: Crystal data and structure refinement for **1**

CCDC number	2449082
Empirical formula	$C_{12}H_{17}Cl_2N_5OPtS$
Formula weight	545.35
Temperature [K]	296(2)
Crystal system	orthorhombic
Space group (number)	$Pna2_1$ (33)
a [Å]	27.2498(14)
b [Å]	5.4740(3)
c [Å]	23.9942(11)
α [°]	90
β [°]	90
γ [°]	90
Volume [Å^3]	3579.1(3)
Z	8
ρ_{calc} [gcm^{-3}]	2.024
μ [mm^{-1}]	18.576
F(000)	2080
Crystal size [mm^3]	0.018×0.038×0.16
Crystal colour	clear red
Crystal shape	needle
Radiation	Cu K_α (λ=1.54178 Å)
2θ range [°]	6.49 to 139.39 (0.82 Å)

Index ranges	$-32 \le h \le 33$<br>$-6 \le k \le 6$<br>$-29 \le l \le 24$
Reflections collected	19182
Independent reflections	6355<br>R_{int} = 0.0987<br>R_{sigma} = 0.1075
Completeness to θ = 67.679°	99.9 %
Data / Restraints / Parameters	6355 / 1040 / 506
Absorption correction T_{min}/T_{max} (method)	0.3805 / 0.7532 (multi-scan)
Goodness-of-fit on F^2	1.060
Final *R* indexes [$I \geq 2\sigma(I)$]	R_1 = 0.0930<br>wR_2 = 0.2540
Final *R* indexes [all data]	R_1 = 0.1030<br>wR_2 = 0.2645
Largest peak/hole [eÅ^{-3}]	2.85/−1.83
Flack X parameter	0.47(6)

Table S2. Selected Bond lengths [Å] and bond angles [°] for **1.**

Atom–Atom	Length [Å]
Pt1–Cl1	2.278(10)
Pt1–S1	2.221(7)
Pt1–N1	2.06(2)
Pt1–Cl2	2.333(11)
Pt1B–Cl1B	2.2875
Pt1B–S1B	2.1766
Pt1B–N1B	2.0847
Pt1B–Cl2B	2.3125
Pt2–S2	2.220(7)
Pt2–Cl3	2.296(10)
Pt2–Cl4	2.261(12)
Pt2–N18	2.08(3)
Pt2B–S2B	2.2542
Pt2B–Cl3B	2.2811
Pt2B–Cl4B	2.2932
Pt2B–N18B	2.0419

Atom–Atom–Atom	Angle [°]
Cl1–Pt1–Cl2	170.8(6)
S1–Pt1–Cl1	90.8(4)
S1–Pt1–Cl2	94.5(4)
N1–Pt1–Cl1	85.8(9)
N1–Pt1–S1	171.4(8)
N1–Pt1–Cl2	89.8(9)
Cl1B–Pt1B–Cl2B	174.8
S1B–Pt1B–Cl1B	91.4
S1B–Pt1B–Cl2B	93.7
N1B–Pt1B–Cl1B	85.0
N1B–Pt1B–S1B	164.2
N1B–Pt1B–Cl2B	89.9
S2–Pt2–Cl3	92.1(4)
S2–Pt2–Cl4	92.6(4)
Cl4–Pt2–Cl3	175.2(5)
N18–Pt2–S2	174.1(11)
N18–Pt2–Cl3	88.2(11)
N18–Pt2–Cl4	87.0(11)
S2B–Pt2B–Cl3B	93.3
S2B–Pt2B–Cl4B	89.2
Cl3B–Pt2B–Cl4B	175.8
N18B–Pt2B–S2B	169.9
N18B–Pt2B–Cl3B	90.9
N18B–Pt2B–Cl4B	87.2
H34D–C34B–H34E	109.5
H34D–C34B–H34F	109.5
H34E–C34B–H34F	109.5

Isomerisation of 1

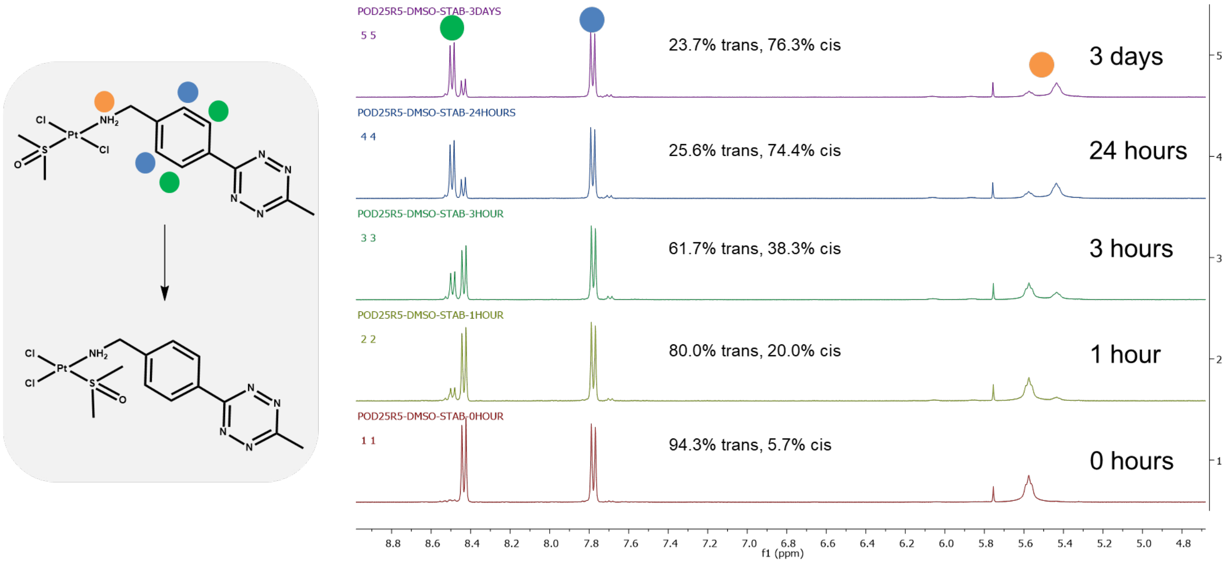

Figure S6. Monitoring the *trans-/cis*-isomerisation of **1** in DMSO-d_6 using ^{1}H NMR.

Stability of 1

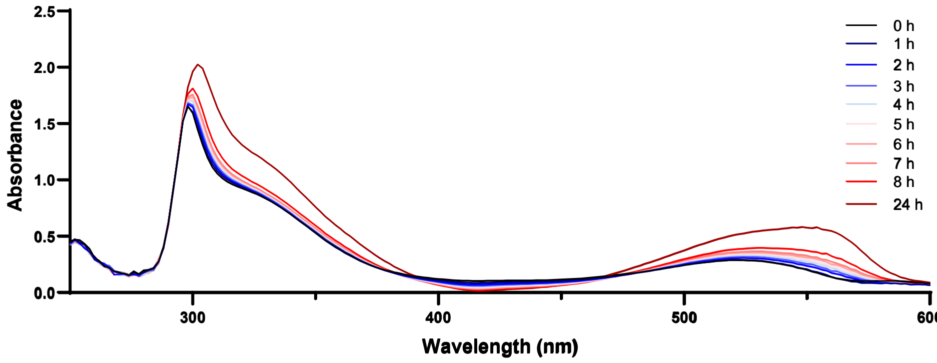

Figure S7. Monitoring the stability of **1** using UV-Vis. UV-Vis spectra of **1** (500 μM) in DMEM:DMSO (200:1) over the course of 24 h at 37 °C.

Pt-tetrazine 1 IEDDA Reactions:

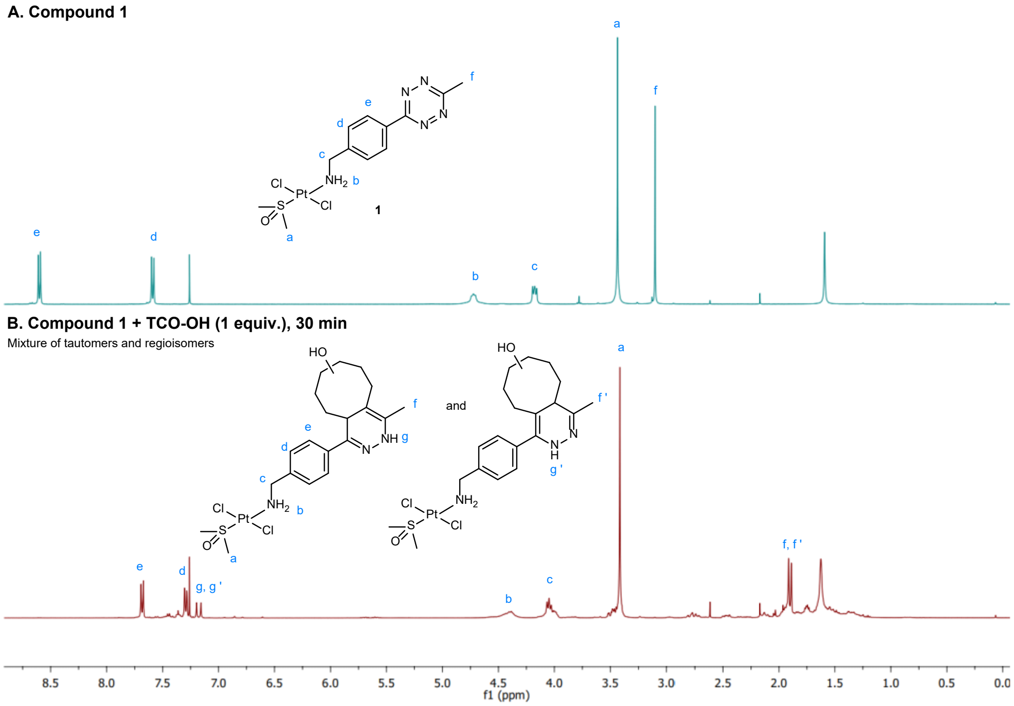

Figure S8. 1 (5.00 mg, 1 equiv.) was dissolved in $CDCl_3$ (0.2 mL) and then TCO-OH (1.16 mg, 1 equiv.) dissolved in $CDCl_3$ (0.2 mL) was added. The mixture was agitated at room temperature for 30 mins and then analysed by ^1H NMR spectroscopy. (a) ^1H NMR spectrum (400 MHz, $CDCl_3$) of compound **1**. (b) ^1H NMR spectrum (400 MHz, $CDCl_3$) of the reaction mixture after IEDDA reaction between compound **1** and (E)-Cyclooct-4-enol (TCO-OH) after 30 min. Important peaks highlighting the formation and the mixture of tautomers and regioisomers in **2** are labelled.

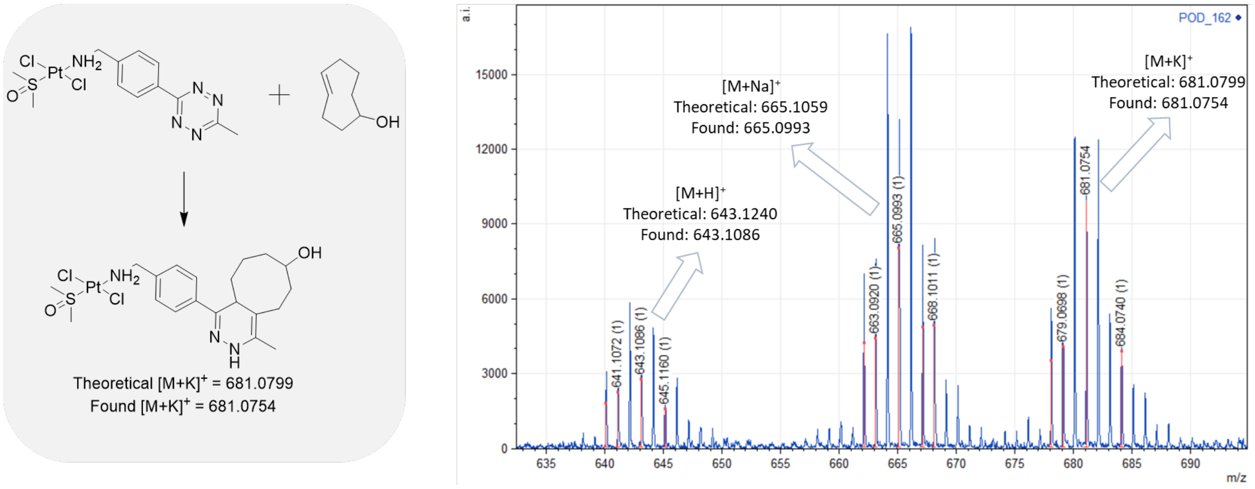

Figure S9. ESI HRMS spectrum of Pt-tetrazine TCO-OH IEDDA reaction mixture highlighting formation of **2** following stirring at room temperature in $CDCl_3$ for 30 mins.

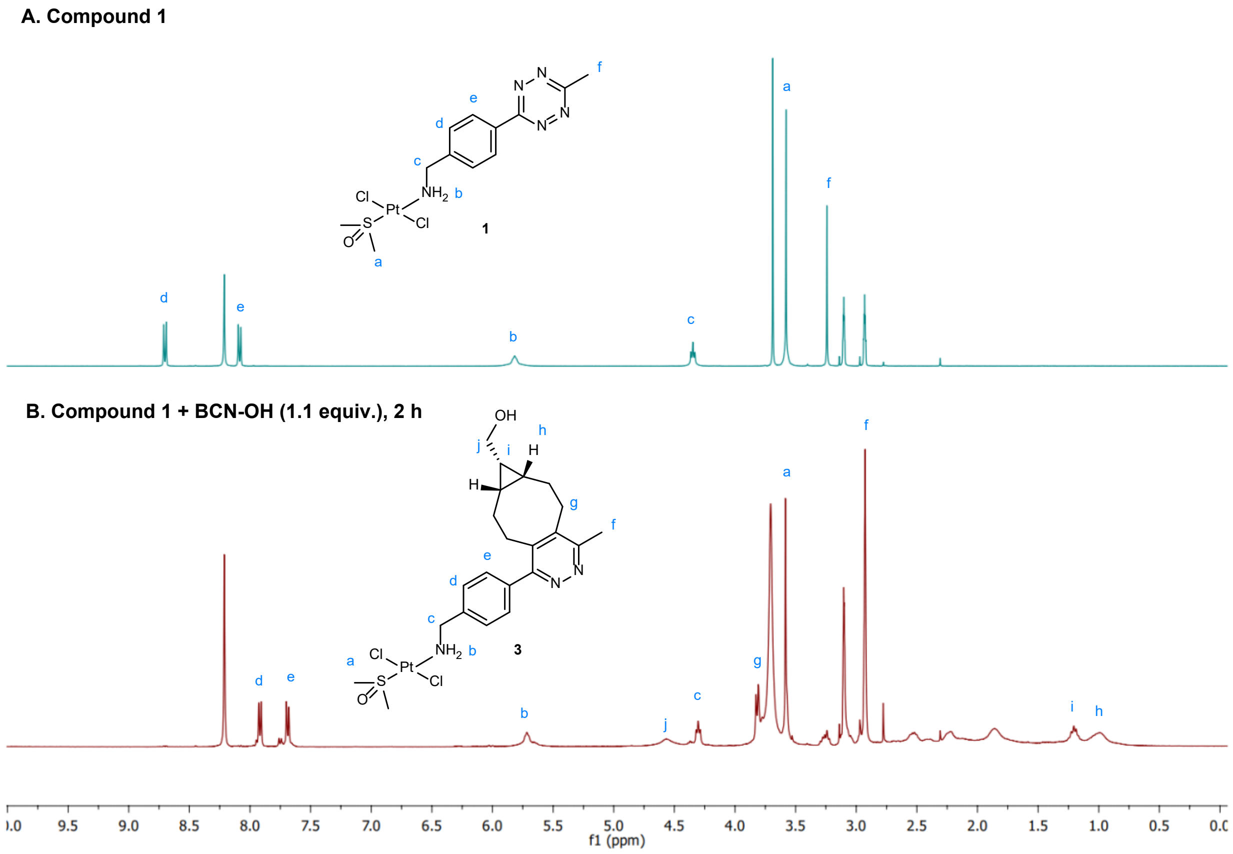

Figure S10. 1 (5.00 mg, 1 equiv.) was dissolved in *N*,*N*-dimethyl-formamide-d7 (0.2 mL) and then BCN-OH (1.51 mg, 1.1 equiv.) dissolved in *N*,*N*-dimethyl-formamide-d7 (0.2 mL) was added. The mixture was agitated at room temperature for 2 hours and then analysed by ^{1}H NMR spectroscopy. (a) ^{1}H NMR spectrum (400 MHz, *N*,*N*-dimethyl-formamide-d7) of compound **1**. (b) ^{1}H NMR spectrum (400 MHz, *N*,*N*-dimethyl-formamide-d7) of the reaction mixture after IEDDA reaction between compound **1** and BCN-OH after 2 h. Important peaks highlighting the formation of **3** are labelled.

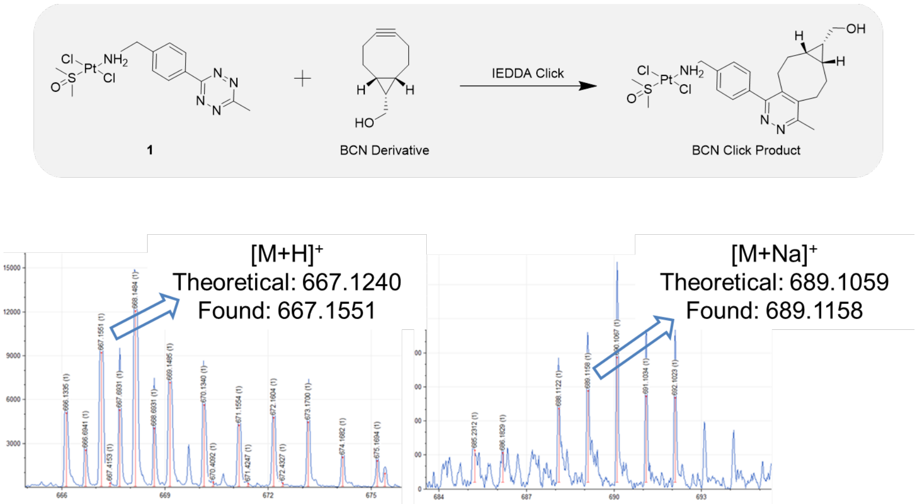

Figure S11. ESI HRMS spectrum of Pt-tetrazine BCN-OH IEDDA reaction mixture highlighting formation of **3** following stirring at room temperature in *N*,*N*-dimethyl-formamide-d7 for 2 hours.

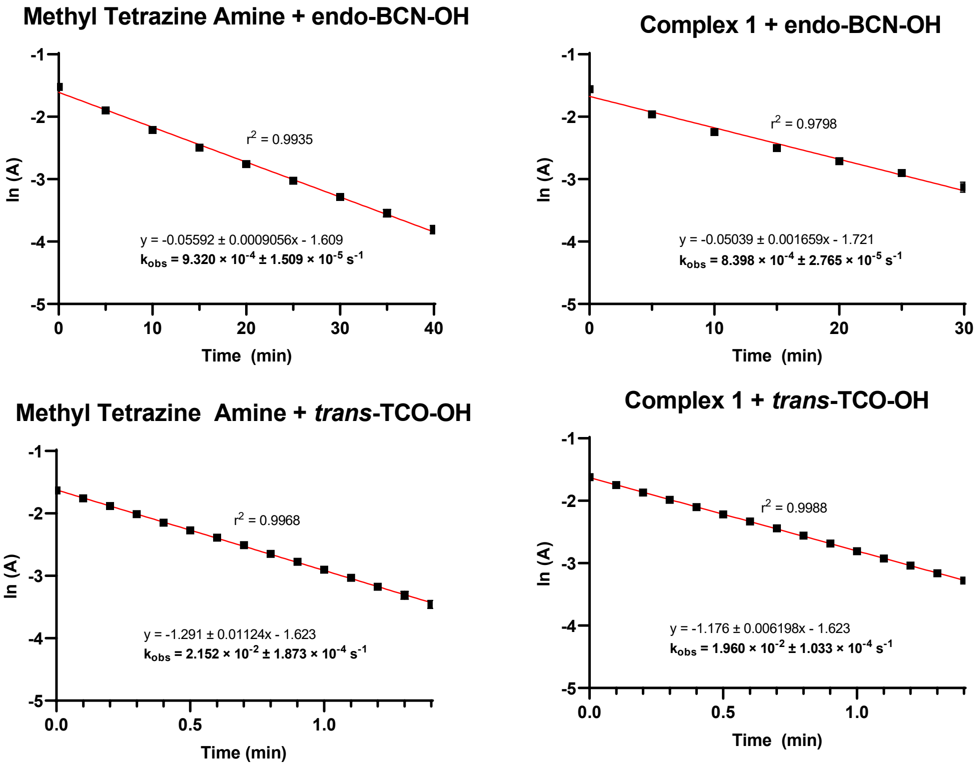

Figure S12. Plots for pseudo first-order IEDDA reactions of (i) methyl tetrazine amine with BCN-OH and TCO-OH and (ii) **1** with BCN-OH and TCO-OH as measured by UV-Vis spectroscopy. The absorbance (A) was measured at 5 min intervals for 60 min for the reaction with BCN-OH and at 6 second intervals for 2 min for the reaction with TCO-OH (n=3).

Table S3. K_{obs} and relative rates of IEDDA reactions of (i) methyl tetrazine amine with BCN-OH and TCO-OH and (ii) **1** with BCN-OH and TCO-OH as measured by UV-Vis spectroscopy (n=3).

**Reaction**	**k_{obs} (s^{-1})**	**Relative Rate***
Methyl Tetrazine Amine + BCN-OH	$9.320 \times 10^{-4} \pm 1.509 \times 10^{-5}$ s^{-1}	1
Methyl Tetrazine Amine + TCO-OH	$2.152 \times 10^{-2} \pm 1.873 \times 10^{-4}$ s^{-1}	23
Complex **1** + BCN-OH	$8.398 \times 10^{-4} \pm 2.765 \times 10^{-5}$ s^{-1}	0.90
Complex **1** + TCO-OH	$1.960 \times 10^{-2} \pm 1.033 \times 10^{-4}$ s^{-1}	21

* Rate of reaction between Methyl Tetrazine Amine + BCN-OH = 1.

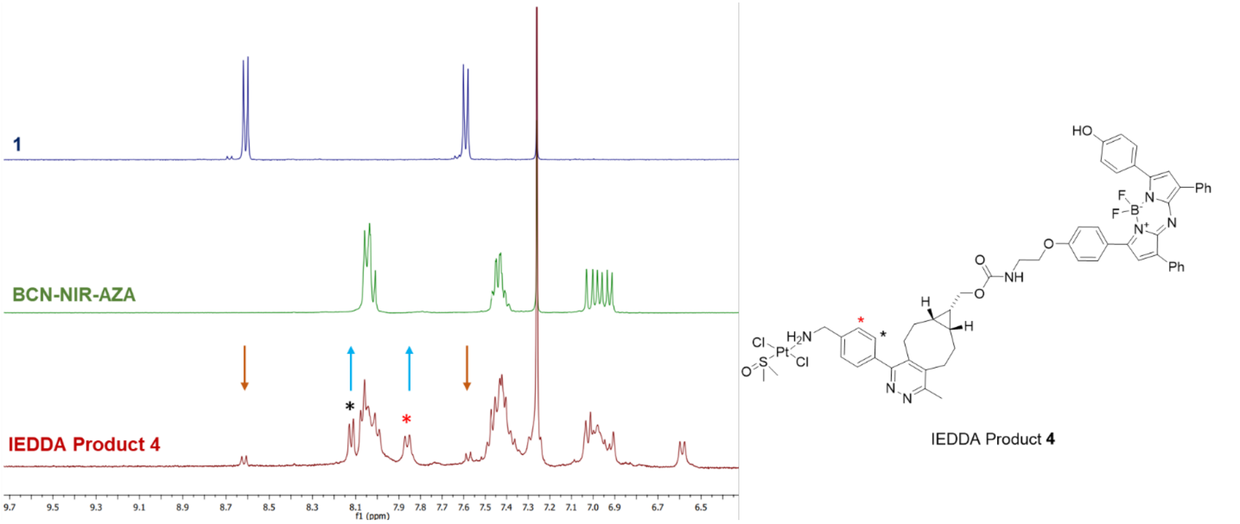

Figure S13. Compound **1** (5.14 mg, 1 equiv.) was dissolved in $CDCl_3$ (0.2 mL) and then BCN-NIR-AZA (7.07 mg, 1 equiv.) dissolved in $CDCl_3$ (0.2 mL) was added. The mixture was agitated for 30 min at room temperature and analysed by ^1H NMR spectroscopy. ^1H NMR of **1** (top), BCN-NIR-AZA (middle), Pt-tetrazine BCN-NIR-AZA IEDDA reaction product **4** (bottom) following stirring at room temperature in $CDCl_3$ for 30 minutes.

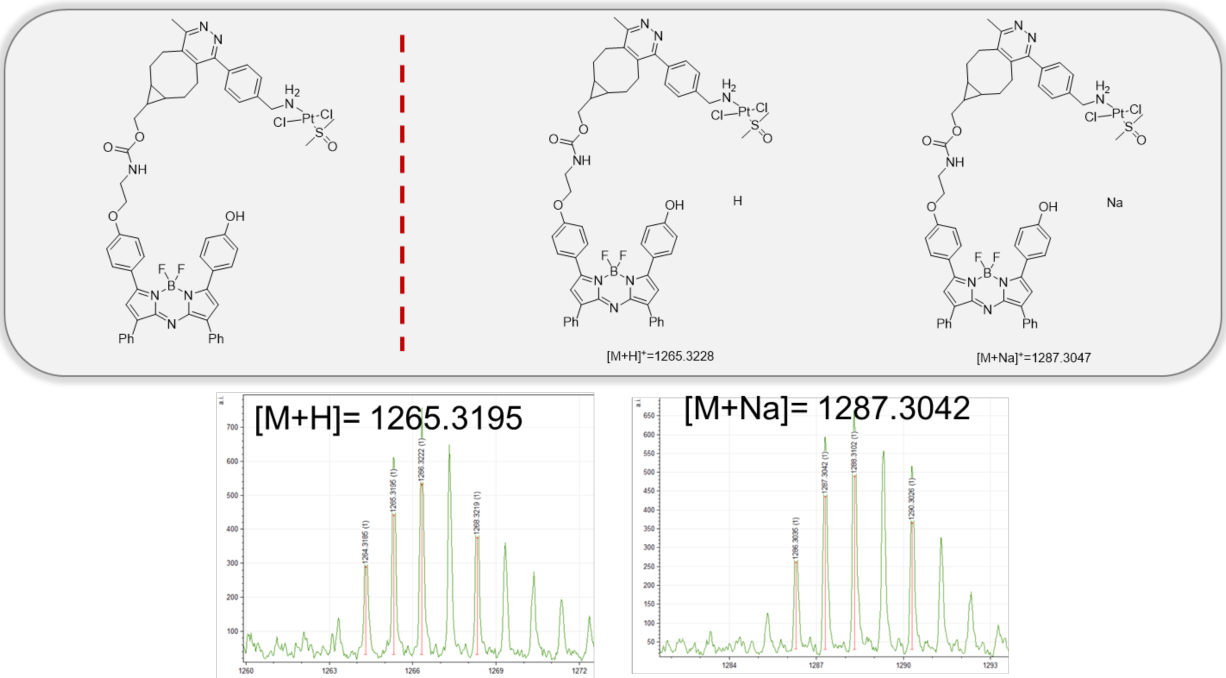

Figure S14. ESI HRMS spectrum of Pt-tetrazine BCN-NIR-AZA IEDDA reaction mixture highlighting formation of **4** following stirring at room temperature in $CDCl_3$ for 30 minutes.

Fluorescence Microscopy:

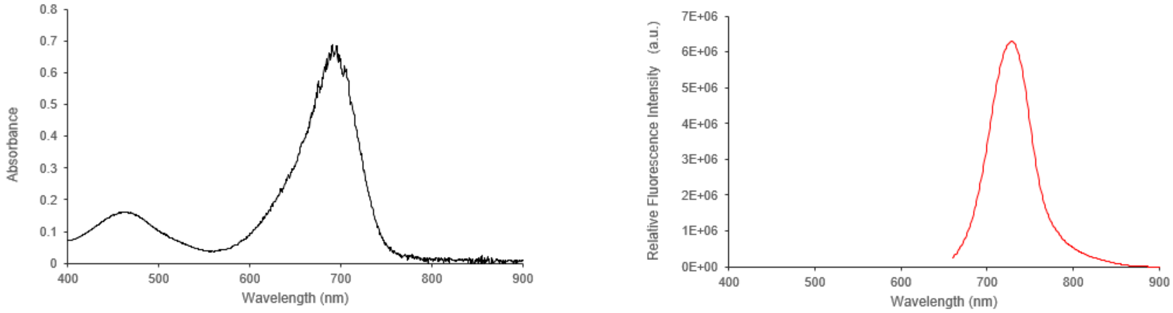

Figure S15. Left: UV absorbance spectrum of IEDDA product **4** (10 μM in $CHCl_3$), λ_{max} abs = 694 nm. Right: Fluorescence spectrum of IEDDA product **4** (10 μM in $CHCl_3$), excitation at 650 nm, λ_{max} flu = 732 nm.

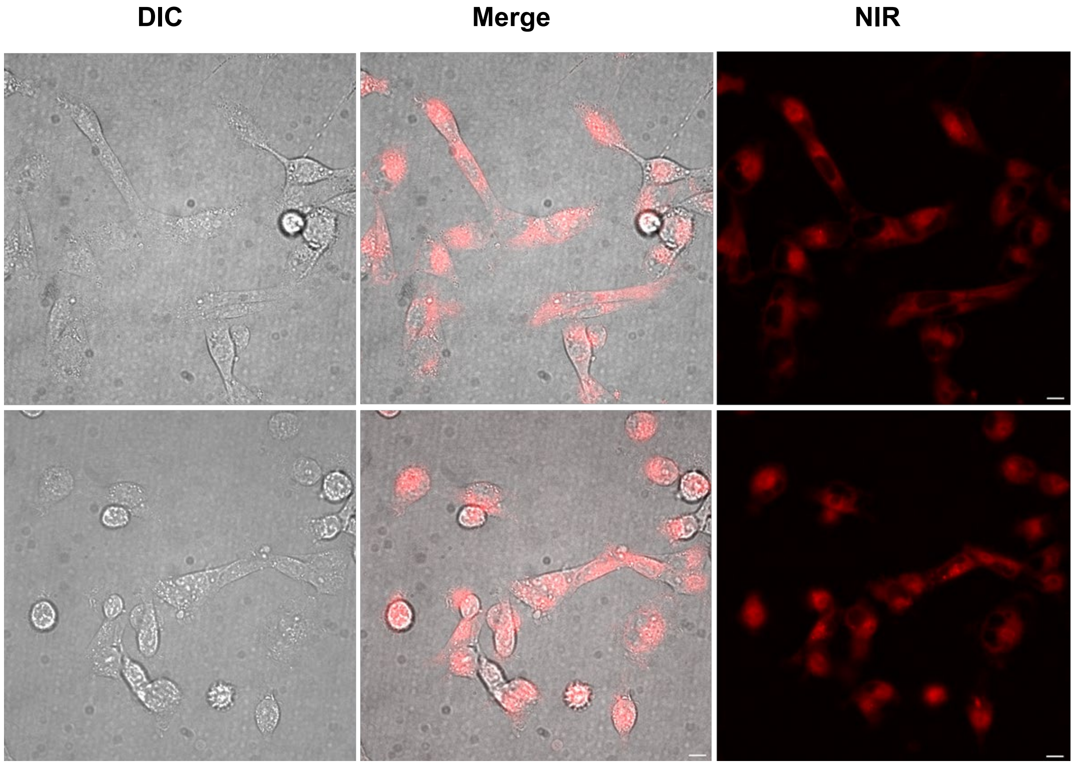

Figure S16. BCN-NIR-AZA in live MDA-MB-231 cell line after 1 hour treatment. Scale bar: 10μm.

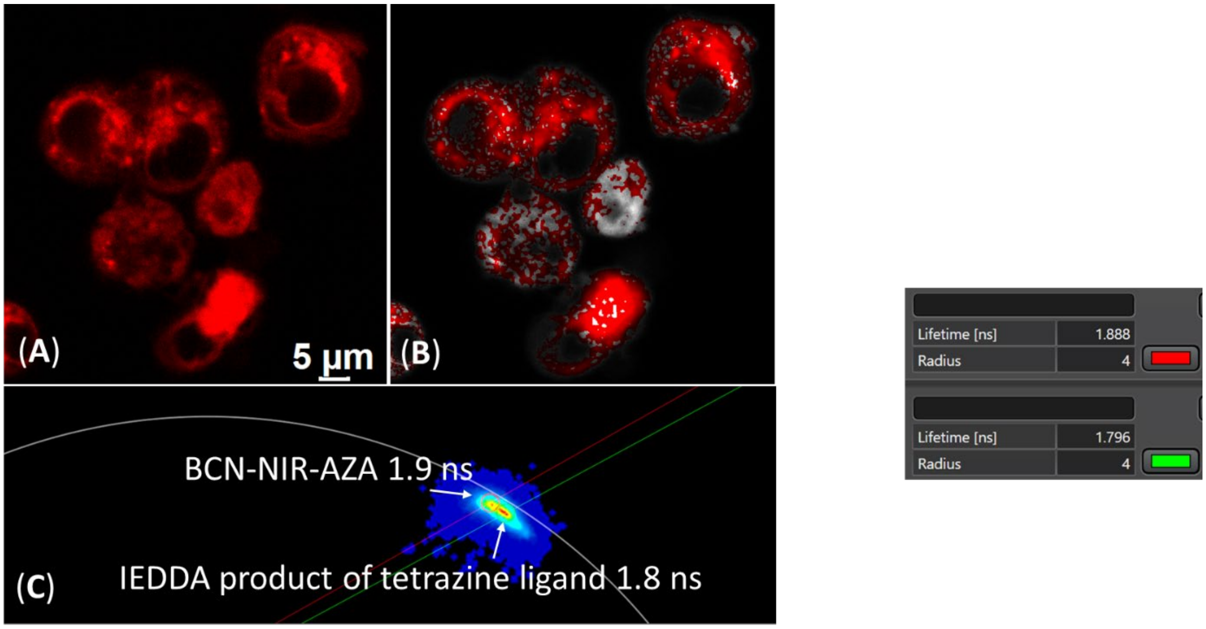

Figure S17. Imaging of IEDDA reaction between methyl tetrazine amine ligand with BCN-NIR-AZA in live MDA-MB-231 cells following 1 hour treatment of methyl tetrazine amine (25 µM) and BCN-NIR-AZA (10 µM). **A.** Confocal image of IEDDA product between methyl tetrazine amine and BCN-NIR-AZA **B.** FLIM Image of IEDDA product between methyl tetrazine amine and BCN-NIR-AZA **C.** Phasor plot of intracellular IEDDA product between methyl tetrazine amine and BCN-NIR-AZA (phase lifetime 1.8 ns) in comparison to BCN-NIR-AZA (phase lifetime 1.9 ns).

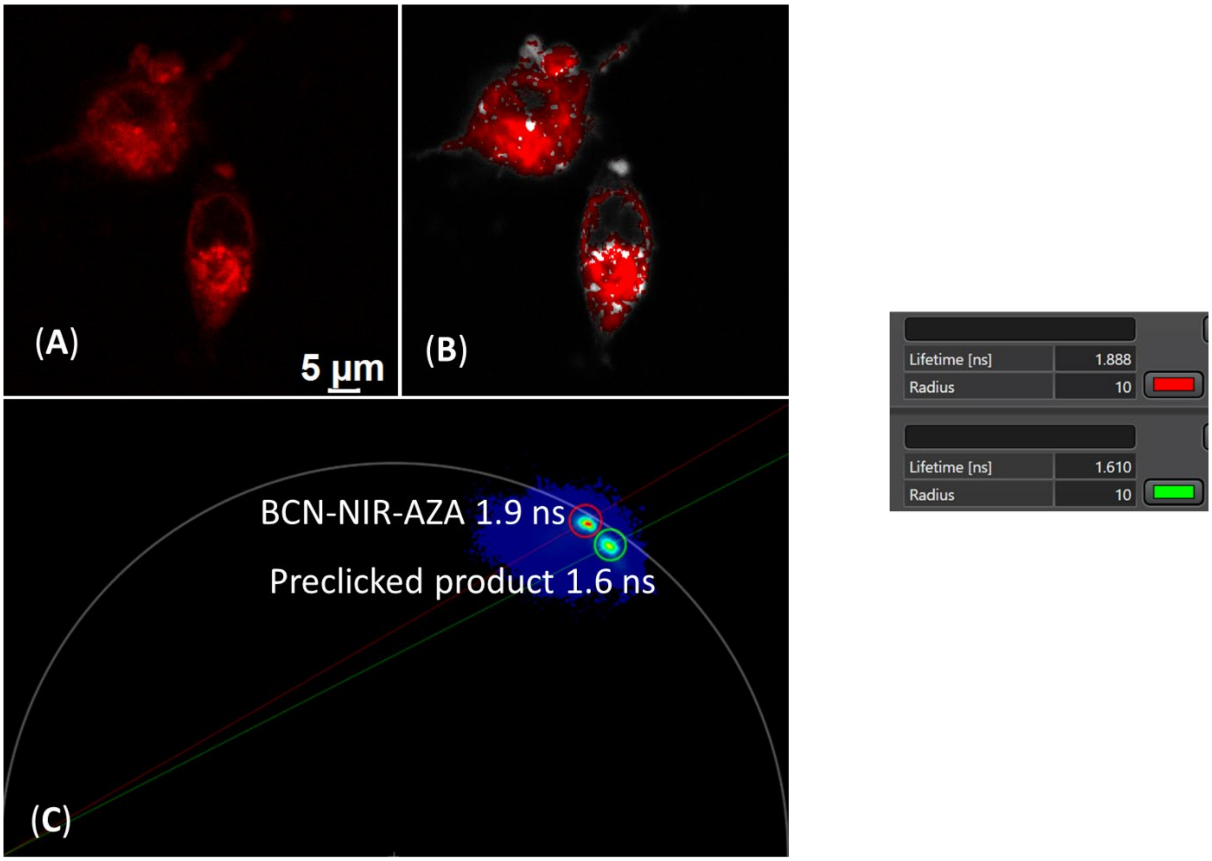

Figure S18. Imaging of preclicked product **4** in live MDA-MB-231 cells following 1 hour treatment of **4** (10µM) **A.** Confocal image **B**. FLIM Image **C**. Phasor plot of intracellular IEDDA product (green circle, phase lifetime 1.6 ns) in comparison to BCN-NIR-AZA (red circle, phase lifetime 1.9 ns).

Table S4 FLIM lifetimes in water.

**Components (0.1 mM)**	**Life time (ns)**
BCN-NIR-AZA	2.45
Preclicked product **4**	2.31
BCN-NIR-AZA + methyl tetrazine amine	2.30
BCN-NIR-AZA + methyl tetrazine amine with 1 mM GSH	2.35
Preclicked product 4 with 1 mM GSH	2.31

References

1. P. Bitha, G. O. Morton, T. S. Dunne, E. F. Delos Santos, Y. I. Lin, S. R. Boone, R. C. Haltiwanger and C. G. Pierpont, *(Malonato)bis[sulfinylbis[methane]-S]platinum(II) compounds: versatile synthons for a new general synthesis of antitumor symmetrical and dissymmetrical (malonato)platinum(II) complexes*, Inorg. Chem., 1990, **29,**645-652.
2. M. Rashidian, L. Wang, J. G. Edens, J. T. Jacobsen, I. Hossain, Q. Wang, G. D. Victora, N. Vasdev, H. Ploegh and S. H. Liang, *Enzyme-Mediated Modification of Single-Domain Antibodies for Imaging Modalities with Different Characteristics.* Angew. Chem. Int. Ed. Engl., 2016. **11;55**(2), 528-533.
3. Bruker, *SAINT*, *V8.40B*, Bruker AXS Inc., Madison, Wisconsin, USA.
4. L. Krause, R. Herbst-Irmer, G. M. Sheldrick, D. Stalke, *J. Appl. Cryst.* **2015**, *48*, 3–10.
5. G. M. Sheldrick, *Acta Cryst.* **2015**, *A71*, 3–8.
6. G. M. Sheldrick, *Acta Cryst.* **2015**, *C71*, 3–8.
7. C. R. Groom, I. J. Bruno, M. P. Lightfoot, S. C. Ward, *Acta Cryst.* **2016**, *B72*, 171–179.
8. D. Kratzert, *FinalCif*, *V151*, https://dkratzert.de/finalcif.html.
